# Successful Treatment of Recurrent Primary Sclerosing Cholangitis after Orthotopic Liver Transplantation with Oral Vancomycin

**DOI:** 10.1155/2013/314292

**Published:** 2013-02-24

**Authors:** Yinka K. Davies, Cynthia J. Tsay, Dario V. Caccamo, Kathleen M. Cox, Ricardo O. Castillo, Kenneth L. Cox

**Affiliations:** ^1^Division of Gastroenterology, Hepatology, and Nutrition, Department of Pediatrics, School of Medicine, Stanford University, Stanford, CA 95762, USA; ^2^Yale University, New Haven, CT 06510, USA; ^3^Department of Pathology, Sutter Medical Center, Sacramento, CA 95816, USA; ^4^Department of Pediatrics, Lucile Packard Children's Hospital, Stanford University, Palo Alto, CA 94304, USA

## Abstract

Primary sclerosing cholangitis (PSC) is a progressive, cholestatic disease of the liver that is marked by inflammation of the bile ducts and damage to the hepatic biliary tree. Approximately 60–70% of patients also have inflammatory bowel disease and progression of PSC can lead to ulcerative colitis and cirrhosis of the liver. Due to limited understanding of the etiology and mechanism of PSC, the only existing treatment option is orthotopic liver transplantation (OLT); however, recurrence of PSC, after OLT is estimated to be between 5% and 35%. We discuss the successful treatment of a pediatric patient, with recurrent PSC, after OLT with oral Vancomycin.

## 1. Introduction

Primary sclerosing cholangitis (PSC) is a progressive inflammatory disease, of unknown etiology and with significant morbidity and mortality, which damages the intra- and/or extrahepatic biliary tree leading to portal hypertension and cirrhosis of the liver. The clinical course is variable including hepatobiliary carcinoma, especially cholangiocarcinoma in 6–20% of patients [1–6]. Additionally, it is estimated that 60–70% of people with PSC have inflammatory bowel disease [7, 8]. Orthotopic liver transplantation (OLT) is the only treatment option for patients with end-stage liver disease due to the progressive damage caused from PSC [9, 10]. The recurrence of PSC in the new graft is estimated to be between 5–35% [11–13]. We report the successful treatment of a patient after OLT, who had shown recurrence of disease, with oral Vancomycin.

## 2. Case Report

**Figure 1:**
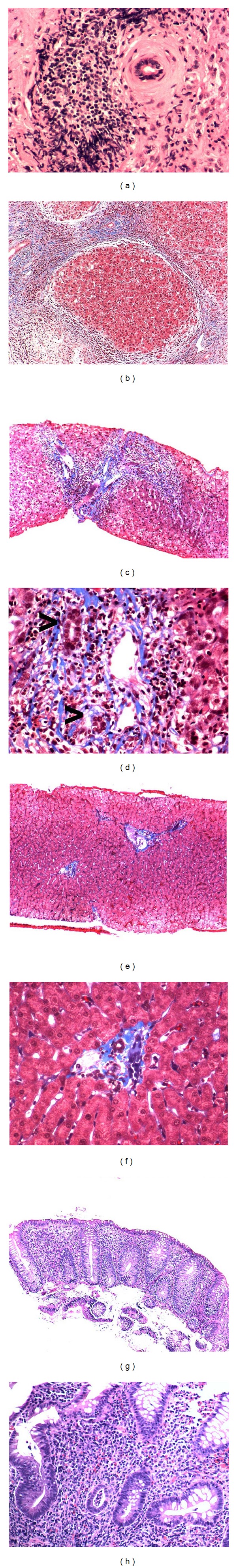
Liver and colon biopsies (stained with trichomere): *thoracic liver before OLT*: (a) onion skin fibrosis and periductular inflammation; (b) cirrhosis nodules surrounded by normal tissue; *after OLT with recurrence of PSC* (c) periductular fibrosis and inflammation; (d) damaged bile ductules noted with arrows; *after  Vancomycin treatment with no immune suppression* (e) normal bile ducts; (f) resolution of portal space ductile damage. Biopsies from (g) descending colon (100x) and (h) sigmoidal colon (200x) show mild irregularities in the contours of the crypts, mild loss of mucin, and evidence of cryptitis with neutrophilic infiltrate within the crypt epithelium.

A 12-year-old girl presented with a three-week history of jaundice and lethargy, with periumbilical pain every other day. There had been no recent travel or sick contacts. Screening tests revealed platelet level was low (89 K/uL; normal range 150 to 400). Antinuclear Antibody (ANCA) was positive with a homogeneous pattern, and Antinuclear Antibody Titer was elevated (640; normal range <40). Serum copper was normal at 1065 ug/L and ceruloplasmin was normal at 24 mg/dL. *γ*-Glutamyl transpeptidase (GGT) level was elevated (139 U/L; reference range from 5 to 36). C-Reactive Protein (CRP) was elevated at 2.80 mg/dL; reference range was from 0.0 to 0.5. PTT was elevated at 48.6 sec; normal range was from 23.3 to 33.8. Prothrombin Time was elevated at 21.0 sec; normal range was from 11.8 to 14.2. INR was increased to 1.9 sec; normal range was from 0.9 to 1.1. Erythrocyte sedimentation rate (ESR) was elevated at 107 mm/hr; bilirubin was elevated at 3.5 mg/dL, and ammonia was elevated at 55 umol/L. Patient underwent an open liver biopsy, which demonstrated the hepatic parenchyma being completely replaced by extensive compartmentalizing fibrosis and regenerative nodules. There was marked bile duct proliferation in the fibrotic areas. The fibrous septae showed a dense inflammatory infiltrate that consisted predominantly of small lymphocytes admixed with plasma cells and neutrophils. There was prominent interface hepatitis at the periphery of the inflamed septae. There was also a component of acute cholangitis showing focal bile duct infiltration and destruction by inflammatory cells, but most of the bile ducts were intact. There was minimal intrahepatocytic cholestasis, but no significant cholestasis was present in the large bile ducts (Figures 1(a) and 1(b)). She underwent colonoscopy and colonic biopsies which demonstrated focal cryptitis, occasional crypt abscesses, and some crypt distortion, mainly in the descending and sigmoid colon, correlating with her serologic marker ANCA, elevated at 640 (Figures 1(g) and 1(h)) consistent with ulcerative colitis. The patient was diagnosed with primary sclerosing cholangitis with inflammatory bowel disease (ulcerative colitis).

She underwent an orthotopic liver transplant three months later with postoperative complications consisting of hypertension. She was discharged home on postoperative day ten. Her postoperative medications included Prograf (Tacrolimus), CellCept (Mycophenolate), Prednisone, Actigall (Ursodiol), Magnesium, Septra (Trimethoprim and Sulfamethoxazole), and Valcyte (Valganciclovir). Over the next three years she did well weaning off everything except Prograf and CellCept. On routine labs (three years after transplant) she was found to have an elevated alkaline phosphatase level (204 U/L; normal range from 50 to 136). Her aspartate aminotransferase (AST) was elevated (267 U/L; normal range from 15 to 37). Her alanine aminotransferase (ALT) was elevated (290 U/L; normal range from 30 to 65). Her GGT was also elevated (309 U/L; normal range from 5 to 55). Her sedimentation rate was elevated (25 mm/hr; normal range from 0 to 20). EBV and CMV titers were negative and she had a therapeutic Prograf level of 7.9.

The patient underwent liver biopsy to distinguish between liver rejection or recurrence of primary sclerosing cholangitis. The transplanted liver biopsy demonstrated prominent portal inflammation consisting of large numbers of plasma cells and lymphocytes and rare neutrophils and eosinophils. There was prominent interface inflammation with occasional acidophil bodies near the limiting plate. There was mild to moderate bile ductular proliferation with very prominent lymphocytic infiltration of the bile ducts. Some of the bile ductules showed branching and there was concentric inflammation and “onion skin” fibrosis around bile ductules. Overall, the lobular parenchyma was unremarkable. There was lymphocytic endothelialitis. Trichrome stain demonstrated little periportal fibrosis and no evidence of bridging fibrosis. Iron stain was unremarkable. The biopsy was compatible with recurrent primary sclerosing cholangitis (Figures 1(c) and 1(d)). The lack of eosinophils in the portal infiltrate was more consistent with recurrent disease rather than acute rejection. 

**Figure 2:**
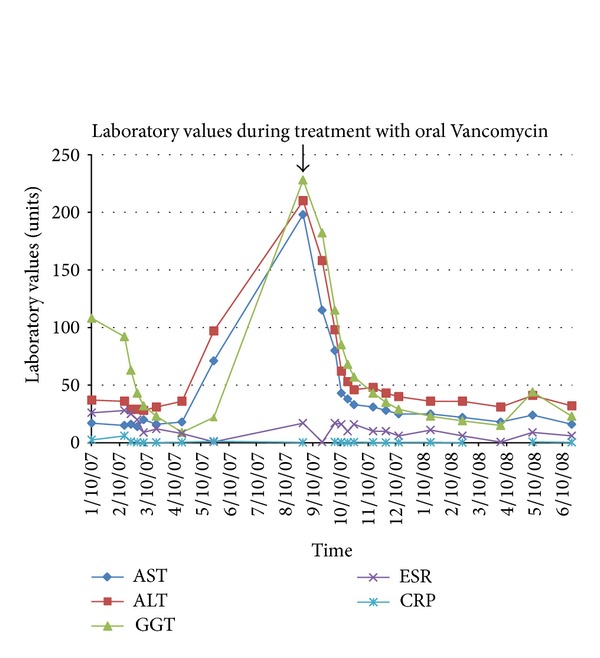
Blood liver markers (AST, ALT, GGT, ESR, CRP) returned to normal values after treatment with oral Vancomycin was started in August 2007 (see arrow). To this date, she remains on oral Vancomycin with normal laboratory values (AST: 15–37 U/L, ALT: 30–65 U/L, GGT: 5–36 U/L, ESR: 0–20 mm/hr, CRP: 0.0–0.5 mg/dl).

**Figure 3:**
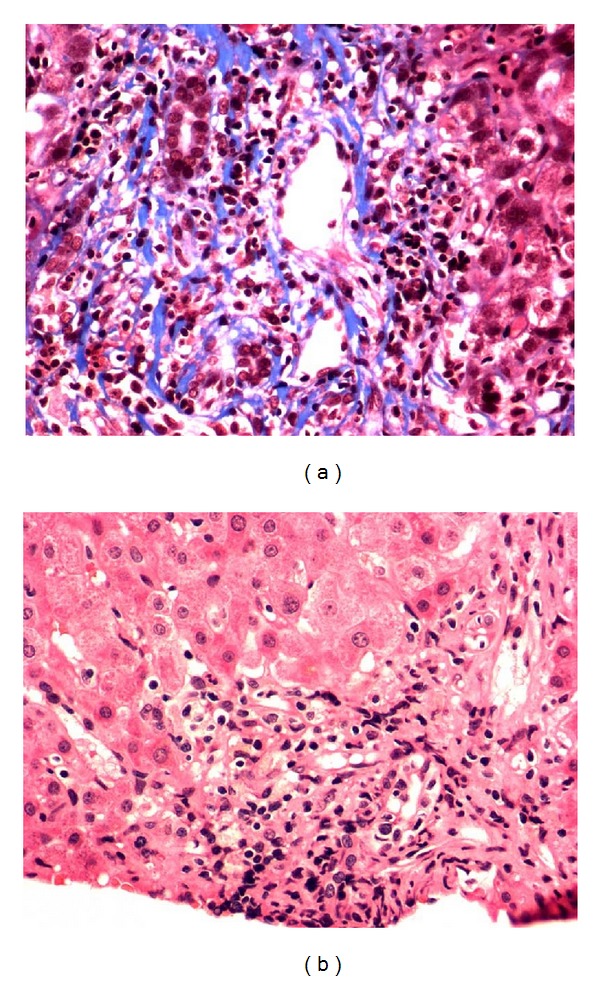
Comparative liver biopsy of bile ductules (a) before Vancomycin (recurrent PSC) and (b) after Vancomycin. Resolution of inflammation and cirrhosis is visible.

She was treated for recurrent primary sclerosing cholangitis. Her immunosuppressant medication remained the same and oral Vancomycin was added to the regimen. She received 500 mg orally three times a day of Vancomycin. During the course of her treatment with oral Vancomycin, liver enzymes (AST, ALT, GGT, ESR, and CRP) returned to normal values (Figure 2). The patient is currently on Vancomycin and remains well, to date, with normal laboratory values. Her repeat liver biopsy after three years from the recurrence of her primary sclerosing cholangitis showed no evidence of onion skin around the large bile duct and no inflammation of the bile ducts (Figures 1(e) and 1(f)). Comparison of biopsy photos before and after Vancomycin reveals a return to normal liver structure and anatomy (Figure 3). 

## 3. Discussion

In this paper, we describe the successful use of oral Vancomycin in the treatment of recurrent PSC, after OLT. Currently, there is no universally accepted treatment for PSC; progressive cirrhosis of the liver inevitably leads to the need for liver transplantation in most patients. The majority of available therapeutic options, such as immunosuppressants, corticoid steroids, and ursodeoxycholic acid (UDCA), deal with complications resulting from PSC and do not inhibit progressive damage from the disease itself [14]. Studies have demonstrated that while UDCA can lead to improvements in biochemical markers, the potential for PSC recurrence requiring OLT is relatively high [15, 16]. Therefore, liver transplantation is the only life-extending option for individuals with advanced stage liver disease. Most data regarding PSC deals with adult cases, and some evidence has shown that the course of the disease in adolescents may follow a unique clinical course, notably an elevation in autoimmune features such as elevated antinuclear antibodies [17]. While juvenile PSC is rare, it contributes to around 2% of pediatric LT annually. The limited number of effective treatment options is primarily due to the unknown pathogenesis of PSC, although some speculate that the primary disease agent is from an enteric bacterial infection.

Previous studies have demonstrated that oral Vancomycin is a viable treatment for early stage PSC, before extensive cirrhosis of the liver is present [18, 19]. An extended clinical study demonstrated that long-term treatment with oral Vancomycin in fourteen juvenile PSC with IBD cases led to the normalization of liver blood markers and reversal of histological abnormalities. However, relapse in GGT and ALT occurred after treatment was suspended [19]. Oral Vancomycin is an immunomodulating bacteria glycopeptide antibiotic that is used to treat gram-positive infections with staphylococcal and has been shown to be poorly absorbed systemically [20, 21]. Vancomycin could also be acting as an anti-inflammatory agent, suggesting that PSC could be an autoimmune disease.

The successful use of oral Vancomycin in reversing the effects of early stage PSC can help develop current understanding regarding the mechanism of the disease. Ongoing clinical trials can potentially lead to new information in regard to the etiology and pathology of the disease. Specifically, this case demonstrates that oral Vancomycin was effective in a case of recurrent PSC after OLT, suggesting that the disease mechanism is not confined within the liver and has external causes—potentially from the backflow of gut bacteria or the spread of gut bacteria through the blood. However, larger cohorts of children with PSC are needed for a more thorough, longitudinal investigation regarding the mechanism, pathogenesis, and treatment of juvenile PSC.
